# Supplementary material for: Are carbon emissions trading and green financial instruments synergistic? -Comprehensive quantitative research based on content analysis
Source: PLoS One. 2024 Mar 7;19(3):e0298601. doi: 10.1371/journal.pone.0298601 (PMC10919601; doi:10.1371/journal.pone.0298601)
Supplement: S1 Data — (ZIP) [file pone.0298601.s001.zip › Data/地级市Z_F工作报告.zip!/║ú─╧/╚2╤╟╩╨/└┤╫╘╣1⁄2╓┌║┼í╛┬φ┐╦╩2╛▌═°í┐_2009.docx]

各位代表：

现在，我代表市人民政府向大会报告工作，请予审议，并请市政协各位委员和其他列席人员提出意见。

一、2008年工作回顾

2008年是海南建省办经济特区20周年，是三亚发展进程中极不平凡的一年。这一年，在市委的正确领导下，全市上下团结一心，奋力拼搏，积极应对挑战，坚持科学发展，克服了诸多不利因素的影响，全市经济保持了平稳较快发展的态势，社会事业全面进步，人民生活进一步改善，取得了来之不易的发展成绩，完成了市五届人大三次会议确定的年度任务。

过去的一年，经济总量稳步扩大，发展效益进一步提升。初步核算，全市生产总值完成144.3亿元(不含农垦，下同)，同比增长16.8%；人均生产总值30572元，增长14.6%。其中，第一、二、三产业分别实现增加值25.9亿元、52亿元、66.4亿元，同比分别增长10.1%、9%、23.8%。全市地方财政一般预算收入21.1亿元，同比增长61.4%，高于GDP增幅44.6个百分点。全市城镇居民人均可支配收入达到13471元，同比增长15.7%；农民人均纯收入5189元，增长18.6%，六年来首次超过城镇居民收入增幅。金融环境进一步改善，截至去年12月底，全市金融机构本外币各项存款余额280.2亿元，比去年年初增长25.7%；贷款余额95.3亿元，比去年年初增长12.6%。金融机构整体盈利6.9亿元，同比增长87.2%；不良贷款率为2.4%，比去年年初下降了11.9个百分点。

过去的一年，重点项目顺利推进，投资规模继续扩大。进一步简化了投资审批程序，完善了项目进入联审制度，严厉打击违法建设行为，切实落实重大项目责任制，严格实施“两个24小时”和“两个一律”制度，投资环境不断改善。在国际金融危机背景下，抓住中央扩大内需的时机，全力争取新增投资，不断加快项目建设进度，全市投资增速保持稳定，投资质量进一步提升。2008年全社会固定资产投资完成151.6亿元，同比增长38.2%；其中，重点项目完成投资91.5亿元，增长42.2%。三亚丽思卡尔顿酒店、三亚悦榕庄、亚龙湾铂尔曼度假酒店等已竣工营业，东环铁路三亚段、凤凰机场二期、南山港一期、新佳鹿回头、凤凰岛开发、三亚湾新城、凤凰水城、时代海岸等项目总体进展顺利。在海棠湾开发方面，开维丽笙、中港万丽、皇圃、高胜、费尔蒙以及蜈支洲岛中心酒店等项目已陆续进场开工，供水厂、污水处理厂、安置区以及市政道路等基础设施建设也顺利推进。

过去的一年，产业结构进一步优化，发展方式转变不断加快。旅游产业方面，克服了出入境政策调整、国际金融危机等不利因素的影响，积极融入国际旅游岛建设，继续推动旅游国际化进程。借助博鳌论坛期间国事活动、北京奥运圣火三亚首传等活动的效应，加强城市营销和市场开发，深度开展了“清凉一夏、三亚度假”等系列主题促销活动，推出了“香港商务购物+三亚度假”一程多站式旅游产品。旅游规划修编顺利完成，旅游服务标准化工作扎实推进，旅游产业总体保持平稳发展。2008年全市接待过夜游客人数604.2万人次，同比增长12.2%；其中，海外游客51.2万人次，同比下降2%。旅游总收入91.1亿元，同比增长13.7%；其中，旅游外汇收入2.6亿美元，增长7.4%。农业方面，积极推动农业生产标准化、集约化、规模化发展，加快槟榔田洋等设施农业基地以及供港澳果蔬国家备案基地标准化建设，进一步加强农产品质量安全工作，农业现代化水平逐步提高。2008年农业总产值39亿元，同比增长10%。创新创意产业方面，加快编制创意产业发展规划，我省首个中小企业公共技术服务平台及科技产业孵化器已投入运行。创意新城基础设施建设和项目引进工作进展顺利，已经与深圳中兴国际投资有限公司等7家公司签署了项目投资协议，入驻工作有序推进。积极开展省电子信息产业基地招商工作，省动漫产业基地也已在三亚同济科技园挂牌。受创新创意产业发展的刺激，工业发展进一步加快，2008年工业总产值26.6亿元(不含中海油)，同比增长19.5%。商贸业方面，完成了《三亚市商贸产业发展规划》编制工作，积极推进农产品批发市场建设、农贸市场升级改造和“万村千乡市场工程”，城乡商品市场体系进一步完善。2008年全市社会消费品零售总额38.8亿元，同比增长26.5%。（上接一版）房地产业方面，依托独特的资源环境优势，不断优化住房供应结构，积极组织房地产促销活动，在全国房地产市场景气度下降的情况下保持了较为稳定的发展势头。2008年房地产开发投资76.2亿元，同比增长48.3%；各类商品房交易面积109.7万平方米，增长17.6%；房地产业实现增加值16.3亿元，增长32.4%。文化产业方面，《三亚市文化产业发展规划》编制工作顺利推进，文化体制改革取得实质性进展。三亚体育中心项目已经开工，三亚文化中心等设施建设筹备工作进一步加快。积极开展保护知识产权专项整治行动，改善了文化市场环境。成功举办了第25届世界精英模特大赛总决赛、2008世界沙滩排球巡回赛、2008环海南岛国际公路自行车赛三亚公开赛等活动，积极开展城乡文艺汇演、文化下乡等工作，营造了良好的文化氛围。

过去的一年，节能减排力度不断加大，可持续发展能力有所提升。加快了基础设施建设，三亚垃圾填埋场、红沙污水处理厂升级改造项目已投入试运行，鹿回头污水处理厂、市区污水管网改造等项目进展顺利，亚龙湾区域集中供冷节能项目有序推进。认真开展污染源普查工作，大力推动主要污染源在线监控设施建设，进一步加强了项目环评管理。积极开展各项环保专项行动，加大了对工业废气和建筑施工噪声超标的整治力度，严厉查处了一批环境违法企业。积极倡导节能理念，推广实施节能建筑设计标准，完成了公共照明节能改造。2008年，我市化学需氧量、二氧化硫排放量两项指标实现双下降。

过去的一年，城市面貌进一步改善，新农村暨文明生态村建设成效显著。城市总体规划重编工作有序开展，城乡统筹概念规划国际竞赛基本完成，城中村改造总体规划进展顺利。不断加大市政基础设施建设力度，扩建了榆亚大道,对河西路、河东路、金鸡岭路等道路进行了改造，完成了三亚河景观工程以及榕根区污水管道等污水管网建设，鹿回头海景大道、红树林公园等项目进展顺利，天然气输配管网覆盖面进一步扩大。不断强化城市管理，重新调整综合行政执法体制，规范城市公共资源使用审批程序，在全省率先出台了户外广告设置规划和技术标准。积极开展环境综合整治活动，顺利通过了全国爱卫办的“创卫”技术评估。完成了十大新农村片区规划，2008年创建文明生态村68个。农业科技服务“110”体系进一步健全，大隆灌区工程等基础设施建设进展顺利，农村公路通畅工程有序推进，全市新建沼气池3315户，完成了92个自然村的饮水安全工程，民房改造、扶贫攻坚等工作取得新成效，农村生产生活条件进一步改善。继续推进生态市建设，不断强化海域使用管理，加强海洋环境监察，积极开展“蓝丝带”海洋环保等活动，加大植树造林和城市绿化工作力度，生态环境质量继续保持全国一流。

过去的一年，改革创新深入推进，对外开放取得新成绩。国企重组改革、关闭退出等工作稳步推进，企业财务预算管理制度、财务动态监测体系不断完善，企业经营风险法律保障体系逐步建立，国有产权管理得到进一步规范。以增强统筹保障能力和服务民生能力为目标，优化了财政支出结构。城乡宅基地和房屋确权登记发证工作有序开展，农村征地补偿配套制度进一步完善。积极支持和配合农垦改革发展，与省农垦总局签订了全面战略合作框架协议，双方合作取得初步进展。与海口海关签署了紧密合作机制备忘录，口岸建设全面升级，口岸通关服务保障能力进一步提高。2008年接待国际豪华邮轮132航次，邮轮接待量跃居全国内地首位；凤凰机场旅客吞吐量突破600万人次，居全国内地机场第19位。与墨西哥坎昆市等城市结为友好城市，积极发挥香港三亚同乡联谊会等组织和团体的纽带作用，促进了三亚对外交流。大力引进和利用外资，组织参加海南香港经贸活动周等商务活动，2008年实际利用外资1.5亿美元，同比增长15.4%。继续扶持出口企业实行技术改造，2008年实现进出口总值7647万美元，同比增长15%；其中，出口6647万美元，增长17%。

过去的一年，民生事业蓬勃发展，人民群众得到的实惠不断增多。出台了《三亚市2008-2013年重点民生项目发展规划》，大力推进基本公共服务均等化。劳动就业方面，2008年城镇新增就业岗位2.5万个，农村富余劳动力转移1.8万人，城镇登记失业率控制在2%以内。社会保障方面，努力完善社保制度，社保覆盖面进一步扩大。出台了《三亚市被征地农民社会保障奖励暂行办法》，进一步加快了新型农村合作医疗、城镇从业人员和城镇居民基本医疗保险待遇调整。农垦各项社会保险已顺利移交地方管理。全市低保已基本实现动态管理下的“应保尽保”。住房保障方面，积极建立健全住房保障制度，切实落实住房补贴和廉租房实物配租工作，廉租房、经济适用房项目顺利推进。教育方面，市中等职业技术学校二期工程、中小学校舍建设等项目顺利推进，义务教育“两免一补”、中职学校“三免一补”、高中学生免学费等政策得到较好落实。三亚学院三期、琼州学院三亚校区等高等教育项目顺利推进，全市高校在校学生人数已达2.2万人。医疗方面，市人民医院改扩建、中医院新区等项目建设顺利推进，农垦三亚医院医疗区升级改造总体规划一期工程已正式启动。社区卫生服务网络覆盖面继续扩大，行政村卫生室覆盖率已达100%。重大疾病防治工作成效显著，保持了霍乱等传染病疫情零纪录。社会和谐方面，精神文明建设取得新进步，社会治安综合治理成效显著，圆满完成了各项重大活动的安全保障工作。“无毒市”建设稳步推进，三亚戒毒康复农场成为全国示范点。有效应对三聚氰胺事件，食品药品安全保障有力。妥善处理了出租车停运事件，信访、安全生产等工作进一步加强，社会保持和谐稳定。

过去的一年，行政管理体制改革有序开展，政府自身建设进一步加强。全力抓好省里下放行政管理事项的对接培训工作，各管理事项平稳运行，对接效果良好。进行了海棠湾机构改革调整，积极探索赋予海棠湾市一级的行政审批权力。加强政府执行文化建设，大力推行首问责任制、岗位责任制和重大事项跟踪督办制，充分发挥市机关效能投诉中心的作用，严格执行行政过错追究制度和投诉处理机制，行政效能不断提高。认真执行市人大及其常委会的决议、决定，自觉接受人大监督，办理市人大代表建议、批评和意见58件。主动接受政协民主监督，办理市政协委员提案112件。积极加强同各民主党派、工商联、无党派人士和人民团体的联系，虚心听取各界人士的建议、意见。大力开展政府投资项目资金管理使用情况监督检查，2008年共完成237个审计项目，审计处理和责成纠正各类违规资金4.1亿元，为财政增收节支5亿元。积极开展反腐倡廉“制度执行年”活动，切实做好治理商业贿赂工作，2008年共查办各类违法违纪案件34件，为国家和集体挽回经济损失1600多万元。

此外，我市国防动员、民兵预备役工作不断进步，拥军优属、拥政爱民活动深入开展，军政军民团结进一步巩固。民族、宗教、外事、侨务、气象、防震减灾、人口计生、妇女儿童、新闻、统计、档案及修志等工作取得新成绩，工会、共青团、科协等工作进一步加强，红十字、老龄和残疾人等事业也取得了新的进步。

各位代表，在发展形势复杂多变、不利因素明显增多的情况下，过去一年我市的经济社会发展能够取得这些成绩，确实来之不易。这是市委正确领导的结果，是市人大、市政协、各民主党派、工商联、无党派人士以及各人民团体监督支持的结果，是中央和省驻三亚各单位、驻三亚部队、武警官兵、公安司法干警积极参与和配合的结果，是广大海内外朋友关心、支持和帮助的结果，是三亚人民开拓创新、团结奋斗、顽强拚搏、辛勤劳动的结果。在此，我谨代表市人民政府，向所有参与、支持、关心三亚建设和发展的同志们、朋友们表示崇高的敬意和衷心的感谢！

在肯定成绩的同时，我们也清醒地看到，在前进中我们仍然面临着不少困难和挑战，政府工作还存在一些问题和不足，主要表现在以下几个方面：一是经济发展速度有放缓的趋势。尽管去年我市的主要经济指标和产业发展指标仍然保持了增长态势，但国际金融危机的影响已逐步显现，GDP和一些重要的产业发展指标增速明显放缓，海外游客数量等甚至出现负增长。今年的国际国内发展形势仍然存在较多的不确定性因素，我们必须做好面对严峻考验的准备。二是财政收支压力增大。随着国际金融危机影响的蔓延和加深，房地产等重点税源呈现出减收趋势，加上政策性减收增多，今年财政增收的压力不小，而政府投资和民生等刚性支出力度却需要进一步加大，财政收支将面临巨大的压力。三是就业结构性矛盾突出。一方面仍有部分城镇失业人员和不少农村富余劳动力求职较难，另一方面部分企业技术工人和旅游服务人员短缺。今年的就业形势可能会更严峻，必须大力加强就业培训工作，并引导待业者转变观念。四是一些涉及群众切身利益的问题需要进一步解决。征地拆迁方面仍然存在不少问题，部分国企职工生活困难，农民持续增收困难较多，城乡发展差距仍然较大，农村基础设施和社会事业发展滞后。五是城市管理和配套服务亟待完善。城市管理水平与国际性热带滨海旅游城市的要求还有较大的差距，违法建设等行为屡禁不止，城市配套服务还不够完善，影响了城市品位的提升。六是政府自身建设有待进一步加强。政府执行力还不够强，工作效率还不够高，工作作风有待进一步改进，发展环境有待进一步优化。对这些问题，我们将予以高度重视，并采取有效措施在今后的工作中努力加以改进和解决。

二、2009年工作安排

2009年是实施“十一五”规划的关键一年，是既富于挑战又充满机遇的一年。目前，国际金融危机的影响还在进一步加深，社会投资趋于谨慎，三亚所面临的发展形势可能更加复杂，经济增长的压力可能进一步增大。不过，我们也要看到，尽管国际国内发展的不确定因素较多，但我国经济发展的基本面仍然没有改变，中央保增长、扩内需、调结构的一系列政策措施以及海南国际旅游岛建设将为我市发展提供新的机遇。从我市自身情况来看，综合经济实力进一步提升，基础设施日臻完善，产业结构优化不断加快，生态环境优势日益凸现，投资吸引力显著增强，对外开放水平不断提高，发展条件仍是利大于弊，三亚仍然处在一个重要的发展战略机遇期。我们必须坚定信心，全面把握和科学分析国际国内发展形势，切实把思想行动统一到中央对经济形势的分析判断上来，统一到落实科学发展观的要求上来，立足实际，着眼长远，采取有效措施积极应对各种情况，推动三亚的发展事业不断前进。

根据省委、省政府的总体要求和市委的部署，2009年政府工作的基本思路是：以邓小平理论和“三个代表”重要思想为指导，认真贯彻党的十七大和十七届三中全会以及省委五届三次、四次全会精神，深入落实科学发展观，按照市委五届三次全会的战略部署，进一步解放思想，不断创新，努力扩大投资规模，努力转变发展方式，努力加快城乡建设，努力深化改革开放，努力推动基本公共服务均等化，全力以赴保增长，扎扎实实惠民生，促进经济社会又好又快发展。

根据上述思路，综合考虑各方面的因素，2009年我市经济社会发展的主要预期目标是：全市生产总值增长12%；全社会固定资产投资增长18%；地方财政一般预算收入增长14%；全市接待过夜游客人数增长10%；万元GDP能耗下降2.5%；城镇居民人均可支配收入增长9%，农民人均纯收入增长9%；当年新增就业岗位16000个以上，城镇登记失业率控制在3%以内；人口自然增长率控制在14‰以内。为实现上述目标，我们将重点抓好以下几个方面的工作：

（一）千方百计扩大投资规模，拉动经济发展。保持经济平稳较快发展是当前的首要任务，我们要进一步落实扩内需保增长的政策措施，继续加大投资力度，不断提升投资质量。

――全力推进重点项目建设。加快实施2008年新增中央投资项目，重点抓好保障性住房、农村公路、污水处理设施等项目建设，按时、保质、保量完成任务。2009年我市安排重点项目152个，年度投资预期115亿元。要以海棠湾国家海岸、创意新城、鹿回头半岛及海上旅游、绕城高速公路及其连接线、槟榔村5A级乡村游、落笔洞文化旅游等骨干工程为重点，有效推进项目建设，同时确保项目生产安全和工程质量。一是做好项目前期工作。积极解决过去项目建设中的遗留问题，树立政府诚信形象，坚定投资者和群众的信心。抓好城乡宅基地和房屋确权登记发证工作，坚决打击违法建设行为，加大征地拆迁工作力度，切实解决项目落地问题。二是实行项目跟踪服务。按照“两个24小时”和“两个一律”制度的要求，明确项目责任领导和责任单位，坚持重点突出、动态管理，对全市重点项目实行跟踪服务，随时掌握项目进展情况，及时解决项目推进过程中遇到的困难。三是强化项目业主责任。明确项目推进时间节点，督促业主在规定的时间内完成项目阶段性工作，严格执行项目监督和退出机制，确保项目按时推进。

――科学策划政府投资项目。充分利用目前扩大内需的政策环境，以基础设施建设、产业结构调整、民生工程等为重点，积极策划安排一批政府投资项目，进一步充实投资项目储备库，形成“续建一批、新建一批、储备一批”的发展态势。加强项目可行性研究和设计管理，加快项目的包装、孵化和报批工作，积极争取第二批中央和省级新增投资，大力争取国债资金、省级资金支持。进一步加强与金融机构的项目对接，充分发挥市城投公司融资投资平台的作用，加大政府融资力度，争取信贷支持。强化项目业主单位、设计单位和从业单位的责任，确保项目开工后能够顺利进行。加强项目资金和质量监管，严格控制项目成本，确保项目建设质量。

――大力吸引社会投资。通过改善投资环境吸引社会投资，同时着力改善投资结构，提升投资质量。一是不断优化投资环境。尽快建立主动性的项目进入联审制度，进一步理顺从项目进入立项、报建、建设到竣工管理的整个过程，抓好跟踪服务。积极构建社会信用体系基本框架，继续深入开展创建“五星级服务机关”活动，进一步完善投资环境监督评议评价制度。二是充分利用各种外事渠道和商务平台。积极参加“珠洽会”、“厦交会”等大型商务活动，引进一批重大项目，加快推动项目落地。三是努力提升投资质量。以发展规划为指导，充分发挥产业政策和政府投资的引导作用，着力引进投资规模大、技术含量高、环境污染小、经济效益好、产业带动性强的企业与项目。

（二）加快转变发展方式，增强可持续发展能力。把保增长、扩内需、调结构有机结合起来，坚持“高端取向、旅游龙头、六业支撑”的产业发展战略，加快推进经济结构战略性调整。同时要做好节能减排工作，努力转变发展方式。

――提升旅游产业发展质量。以“2009中国生态旅游年”为契机，紧紧围绕国际旅游岛建设，加快实施新修编的旅游规划。一是加强旅游产品开发。精心培育具有海洋特色、民族特色、丰富文化内涵以及较强参与性的旅游产品。深度开发潜水、海底观光、垂钓和海上游等海洋旅游项目，加快三亚湾游艇二期等游艇设施规划建设，继续发展高尔夫旅游。进一步加强景区建设和管理，优化景区周边环境，将南山、大小洞天、天涯海角等打造成精品景区。二是不断完善旅游服务。加快推进海棠湾高星级酒店群、凤凰岛、半岭温泉疗养度假区等高端酒店、度假区建设，提升旅游接待设施档次。加强旅游管理制度化建设，抓好旅游服务标准化工作。针对散客增多的特点，加快游客到访中心、“天涯通”旅游信息服务系统等项目和城市信息化基础建设，利用科技手段提升服务效率。积极构建诚信旅游体系，进一步完善旅游投诉处理机制。切实加强旅游安全建设，营造平安旅游环境。三是增强三亚可进入性。进一步完善和落实航空补贴政策，大力开展香港――三亚一程多站式旅游合作，推动开通日本――三亚直达航线，加快国际邮轮客运中心建设，争取更多的航空公司、旅游包机商和国际邮轮进入三亚或增加运力投入。积极完善口岸服务功能，简化通关手续，不断提高通关服务保障能力。四是加大城市营销和市场开发力度。组团参加莫斯科国际旅交会、香港国际旅交会、中国国际旅交会等国内外高水平旅游展会，有针对性地组织旅游推介会，积极开展“清凉一夏、三亚度假”、“美丽三亚、浪漫假期”、“蓝色心情?三亚生态之旅”等主题促销活动，在俄罗斯、香港等主要客源地设立三亚旅游办事处，邀请国内外媒体、旅行商来三亚采访考察，同时充分利用国内外知名旅行商的营销网络吸引境外高端游客。

――加快热带特色现代农业发展。通过转变生产方式、推动结构调整、完善基础设施、强化产业服务等手段，推动农业现代化进程。一是大力扶持设施农业发展。不断完善和加快推行龙头企业、合作社、农户三方组合的方式，以供港澳果蔬国家备案基地等为载体，积极推进槟榔田洋等设施农业基地建设，大力扶持设施农业向基地化、集约化、标准化生产和规模化经营方向发展。同时，加快推动农副产品加工业发展，延长农业产业链。二是继续推进农业结构调整。积极推动冬季瓜菜、热带水果以及热带花卉等特色优势产业发展，加快田独镇热带兰花种植示范基地等项目建设，提高土地产出效益。充分利用无疫区的有利条件，促进畜牧业健康发展。三是进一步加强农田水利基础设施建设。积极推进大隆灌区工程建设，大隆灌区总干渠要在年内建成。抓紧实施病险水库除险加固工程，力争完成抱古水库及14宗小型病险水库的除险加固建设。四是不断完善农业科技服务。加快推进农业科学城、农业科技服务“110”体系等项目建设，建立新品种新技术示范基地，积极推广测土配方施肥、动植物病虫害防控等新型农技。五是抓好农业市场服务。进一步完善农业信息和流通服务工作，加快推进崖城、海棠湾农产品批发市场建设，积极打造功能齐全的农产品物流中心。六是继续强化农产品质量安全保障工作。大力推进市、镇、基地三级监测网络体系建设，建立三亚农产品安全检测中心，配套完善六镇农产品检测流动服务站，加强对农产品、农业投入品和农业环境质量的检测、监控，树立无公害农产品品牌。

――积极培育海洋产业。尽快完成《三亚市海洋功能区划》修编，进一步完善海域使用权公开招标拍卖工作，加快推进东锣岛、西鼓岛等无居民海岛项目开发。推动港门中心渔港尽早开工建设，继续采用“企业+渔民”的方式，以企业服务补给船为龙头，组织渔民跟帮作业，大力发展外海捕捞。加大引进优质水产品种的力度，推广标准化养殖。以优质鱼、虾、贝类和海珍品的精深加工为重点，积极鼓励和支持水产品加工出口龙头企业发展，推进无公害水产品产地和产品认证工作。加紧与中石油海洋工程公司的洽谈协商，推动南海油气资源开发后勤服务支撑基地入驻创意新城。

――努力壮大创新创意产业。尽快完成创意产业发展规划及定位的最终编制，全力加快创意新城基础设施建设。早日启动高科技产业投融资平台运作，进一步完善中小企业公共技术服务平台及科技产业孵化器建设，切实落实项目跟进投资、项目风险投资、贷款贴息、财政扶持补贴、引进人才奖励等各种优惠政策。加快推进已签约进入创意新城的7家公司项目建设，加强与美国亚升技术公司、亚洲太阳科技有限公司、大唐电讯、威盛电子等企业的协商、谈判与合作，积极引进有实力的太阳能光伏电子和微电子项目。继续完善省动漫产业基地规划，加快基地开发建设，促进市高新技术产业园发展。

――大力推动商贸业发展。进一步加快商贸服务体系建设，大力开发地方特色旅游商品，并以国际旅游岛建设为契机，积极推进免税商店落户三亚，构建商品类型丰富、服务功能完善、面向国际消费的旅游商业网络。适时推动镇级商业中心建设，继续推进“万村千乡市场工程”，逐步推行农村连锁经营。加快推进第一农贸市场整体改造，基本完成第二、第三农贸市场、鸿港市场以及金鸡岭、丹洲小区农贸市场改造。进一步完善市场监测制度和市场应急预案，继续加大商贸行业市场秩序整顿力度，加快信用体系建设，努力打造诚信商贸环境。

――保持房地产业健康发展。充分发挥环境比较优势，提高资源利用效率，坚持高标准规划、高标准设计、高标准开发、高标准经营和管理，积极发展高端旅游房地产，推进三亚湾新城及鲁能项目、鹿回头小东海、凤凰水城、时代海岸、金中海以及三亚山水国际等项目建设。不断加强公共配套设施建设，完善城市配套功能，提升城市文化品味，营造宜居环境。加大房地产宣传促销力度，切实落实各项减税、免费政策，并在国家政策指导下探索完善促进房地产消费的系列优惠政策。积极培育和规范二手房市场，形成梯度消费模式。针对三亚实际情况，进一步规范产权式酒店的开发管理。建立健全市场信息系统和预警预报体系，保持房地产业健康发展。

――做大做强文化产业。加快推动《三亚市文化产业发展规划》编制工作，进一步深化文化体制改革，鼓励社会资本投资兴办文化产业。积极推进三亚体育中心、三亚大剧院、南中国海影视文化生态园、三亚国际传媒中心等基础设施和产业项目建设，继续引进有实力、有特色的大型文化产业项目。加快推行“政府支持、企业主导、市场运作”的方式，引进、组织和培育精品文化节庆赛事活动。大力开展非物质文化遗产工作，积极打造天涯文化源地。进一步加强对落笔洞遗址等历史文物的保护和利用，加大崖城“中国历史文化名镇”建设力度，加强对黎、苗、回等少数民族文化的挖掘、保护、开发、利用，争取建设一个有规模、有特色、有品位的黎族文化大观园。

――进一步加强节能减排工作。全力加快基础设施建设，鹿回头污水处理厂要建成投入运行，红沙污水处理厂中水回用系统、海棠湾污水处理厂、亚龙湾区域集中供冷节能项目建设要加快推进，三亚湾新城、创意新城、田独、红塘湾、荔枝沟落笔洞区污水处理厂要尽快开工建设。进一步健全我市节能减排相关规定，制定固定资产投资项目节能评估和审查的具体实施办法。严格执行节能减排目标责任制，强化节能降耗和污染物减排指标约束，继续做好污染源普查工作和主要污染物减排核算，进一步加强对各污水处理设施的监察、监测，确保稳定运行、达标排放。关闭实心粘土砖厂，坚决淘汰落后生产能力，对橡胶加工厂和小矿山(点)进行集中整治。加快发展城市公共交通，加强公共机关节能工作，大力倡导节能理念。继续推行节能建筑设计标准，鼓励太阳能、沼气等能源利用。

（三）加快城乡建设步伐，夯实三亚发展基础。积极推动城乡一体化建设试点工作，在拉动投资增长的同时，不断完善城市功能和农村发展条件，为未来的发展打下基础。

――加强规划编制工作。积极推进城市总体规划重编和城乡统筹规划综合深化工作，进一步加快崖城镇总体规划修编及崖城历史文化名镇保护规划、天涯镇总体规划修编及天涯海角景区详细规划，加快推进大东海、两河沿岸、行政中心区等城市设计，基本实现新农村规划全覆盖。

――加快城市建设步伐。要通过拓展城市发展空间，完善城市配套功能，提升城市管理水平，不断优化城市发展条件。一是拓展城市发展空间。进一步加强土地储备和管理，科学调控土地供应，合理配置土地资源，为城市发展服务。全力推进海棠湾国家海岸、创意新城基础设施以及绕城高速公路及其连接线建设，为东、西、北战略拓展打好基础。把旧城区改造和新城区建设有机结合起来，积极推进鹿回头、阳光海岸、榕根、月川、临春、东岸等片区开发，提升城市中、南区域建设水平。二是完善城市配套功能。加快凤凰机场二期、南山港一期等重大交通基础设施建设，加快汽车总站、公交总站建设前期准备工作。尽快完成新风桥扩建、三亚大桥重建等项目的设计准备工作，继续推进鹿回头海景大道、市区污水管网改造、城市电网、天然气输配管网等项目建设。大力推广城市公共资源市场化配置，鼓励有偿、有序使用城市公共资源，转变城市经营方式。三是提升城市管理水平。加强综合整治，严厉打击违法建设、非法营运等行为，积极开展市容环境整治、创卫整改等行动，确保通过国家卫生城市考核鉴定，实现创卫目标。转变管理理念，在加大整治力度的同时着力完善城市交通等公共服务，切实落实住房报建制度，努力提升市民文明水平和法律素养，从源头上疏导管理压力。改革管理体制，进一步理顺行政执法体制，积极建立健全城市网格化管理新机制。加快社区建设，强化社区作用，解决社区居委会办公和活动场地问题，积极引导社区中介组织有序发展、规范运作，不断完善城市基层管理和服务体制。优化管理技术，大力推动地理信息系统、电子监控网络等技术应用。

――积极推进新农村暨文明生态村建设。进一步完善十大新农村片区建设，不断提高文明生态村覆盖率。加快推进十大片区41个创建点的建设工作，其余创建点的建设工作也要迅速展开。高质量改造农村公路通畅工程213公里，新建新农村公路418公里，基本实现所有村民小组主村道硬板化的目标，并以此为基础大力推动农村公交事业发展。加快“一池三改”沼气池建设，力争新增大中型沼气池10个、小型沼气池2400户。尽快解决育才镇生产、生活用水问题，继续推动农村饮水安全、民房改造、农村电网、改水改厕等工程建设，进一步加大扶贫开发工作力度。以各镇区为重点，加快推动农村生活垃圾收集和污水排放设施建设，加强农村环境卫生管理。

――进一步加强生态市建设。尽快完成饮用水源地环境保护规划，继续抓好封山育林、退耕还林、退塘还林、护林防火等工作，加快推进“三边林”工程建设，全面完成1.6万亩海防林建设任务。进一步加强海洋环境保护，强化海洋环境监察，加大重点海湾海洋环境和海岸工程监管力度，加快推进人工恢复与保护三亚湾岸滩工程项目。积极推进创建国家环境保护模范城市工作，着力提升环境监测水平，继续开展环保专项行动，进一步加大对噪声、尾气、扬尘等污染的治理力度。

（四）进一步深化改革开放，增强发展活力和动力。我们要积极应对新的发展形势，把深化重点领域和关键环节改革、提高对外开放水平作为保增长的强大动力，不断创新，进一步加快改革开放步伐。

――当好国际旅游岛建设的排头兵。大力推进国际化改造配套试点工作，积极开展全方位、高层次、宽领域的改革探索和创新。成立市旅游发展委员会，实施“大旅游”管理体制改革。大力支持和规范旅游行业协会发展，建立健全包括游艇协会等分会在内的行业协会体系。与境外客源地知名旅游企业合作，兴办外商独资、外商控股旅行社，并以此推动现有旅行社提升服务水平。将旅游产品、旅游设施等硬件要素和产业政策等软件要素的国际化改造结合起来，通过加快奥林匹克国际村、邮轮母港等项目进度带动政策探索，在国际企业、产品、项目、资本、人才的引进以及邮轮游艇等产业的发展方面积极争取更加开放的优惠政策。

――继续推动经济体制改革。完成59家劣势国企的关闭退出工作，积极引进外来资本参与市属企业改制，加快推进三亚市文化产业发展有限公司组建等工作，培育有实力的龙头国企。进一步健全国企法人治理结构和业绩考核体系，加强财务监管和风险管理，提高国企经济效益。继续实施中小企业成长工程，加大对企业的解困、扶持和服务力度，不断优化企业发展环境。探索建立中小企业信用担保基金，努力解决企业融资难问题。全面落实国家和海南省各项税收、收费优惠政策，切实减轻企业负担。鼓励行业协会和中介组织发展，建立健全相关监管机制。加强市场监管、物价监管，深入开展整顿和规范市场秩序专项行动，维护市场经济秩序。以个别行业为试点探索建立信用管理体系，建立市场各方主体的信用档案，强化市场诚信意识。进一步加强与金融机构的沟通，为推进地方金融机构改革，改善金融服务创造良好条件。继续深化财政体制改革，加强财政绩效预算、评价和审计监督，进一步优化财政支出结构。

――积极推进农村综合配套改革。全面开展集体林权制度改革，进一步加强土地承包经营权流转探索、管理和服务，通过搞活流转发展多种形式的适度规模经营。积极探索推动农村金融机构发展，加快农村信用社改革步伐，力争尽快设立村镇银行和贷款公司、农村资金互助社以及小额贷款公司等新型农村金融机构。不断完善农村信贷担保体系，推行小额信贷、农户联保贷款等微型金融服务，切实落实农民小额贷款贴息、农民贷款风险补偿等各项补贴政策。进一步完善农业保险，落实农业保险保费补贴，提高农业抗风险能力。继续探索和完善既有利于加快发展又能充分维护农民利益的征地制度。

――大力支持农垦改革发展。努力从规划的高度将地方与农垦发展结合起来，支持农垦参与海棠湾、崖城以及城市近郊区建设发展，充分发挥双方的比较优势，力求实现最大程度的双赢。进一步做好农垦各项社会保险移交地方管理的服务工作，支持农垦三亚中专、农垦三亚中学重组以及农垦三亚医院升级改造，支持垦区农村公路、电网、沼气、安全饮水等设施建设。

――积极开展对外贸易。进一步扩大对外开放，积极参与泛珠三角区域合作，继续加强与长三角、环渤海等区域的交流合作。进一步树立口岸为贸易服务的理念，加强口岸建设，加快发展口岸经济，打造一流口岸城市。大力引进具有较强创汇能力的企业，扶持龙头企业加快发展。继续实施科技兴贸战略，支持出口企业技术改造，做好企业出口退税、中小企业国际市场开拓资金等的申报工作，进一步优化外贸发展环境。重点发展反季节瓜菜、水果、花卉、海水产品等热带优势农副产品及其深加工产品，加快实施出口市场多元化战略，建立健全外贸预警机制，努力防范、规避和化解贸易风险。

（五）加快推进基本公共服务均等化，大力改善民生。改善民生是发展的根本目的，也是扩大内需的重要措施。我们要加快实施《三亚市2008-2013年重点民生项目发展规划》，努力为人民群众办好事实事。

――加快住房保障体系建设。加大廉租房、经济适用房建设力度，切实解决困难群众和中低收入群体的住房困难。今年要投资6.8亿元，新建廉租房2145套、12.8万平方米，经济适用房2131套、17万平方米。积极推进民族地区茅草房和危房改造，提高住房公积金缴交比率，力争达到10%。

――大力发展教育事业。不断完善义务教育基础设施，大力推进中小学校舍建设，积极做好经济困难家庭子女和进城务工人员子女接受义务教育工作。进一步加大农村学校投入力度，建立健全农村义务教育经费保障机制。加快推进农村学校布局结构调整，继续开展城镇学校和农村薄弱学校对口帮扶工作。适当提高农村地区尤其是边远地区教师待遇，稳定农村教师队伍。以义务教育阶段规范化学校创建为抓手，全面提升基础教育质量。进一步扩大优质高中教育规模，强化薄弱高中建设，缩小校际差距，提升高中教育水平。加快完成市中等职业技术学校二期工程建设，继续推进“教育移民（扶贫）”工程，进一步扩大“三免一补”政策覆盖面，力争中职在校生达到1万人。继续支持高等教育发展，推进三亚学院三期、琼州学院三亚校区、三亚理工职业学院、长江商学院三亚校区等院校建设。

――提高医疗卫生服务水平。加快推进市人民医院改扩建二期工程、中医院新区二期工程建设，积极推动市妇幼保健院、市疾病预防控制中心整体搬迁工作。加快建设解放军总医院海南分院，打造与国际旅游城市相配套的高水平医疗、保健、康复机构。加强社区卫生服务中心、卫生服务站建设，力争社区卫生服务覆盖率达到100%。建立健全社区卫生服务运行机制，积极推动社区首诊制度试点。继续完善以中心卫生院为重点的农村卫生服务体系，积极开展卫生院标准化建设，配套基本医疗设备，继续开展城市医疗机构对口支援卫生院工作，提高农村卫生服务水平。进一步规范药品和医疗器械采购行为，科学确定限价病种、收费标准，完善医疗收费及药品价格公开和查询制度，着力解决“看病贵”问题。继续加强出生人口性别比偏高问题综合治理，稳定低生育水平。

――加强就业再就业工作。充分发挥市人力资源市场的作用，加强就业服务信息网络建设，完善就业登记制度，建立健全城乡一体化的市、区镇、村（社区）三级公共就业服务体系，全面开展公共就业服务。倡导创业带动就业，建立创业培训基地，大力实施资助性创业培训，切实落实创业贷款优惠等政策。积极引导和鼓励高校毕业生面向基层就业，加快公益性岗位开发，继续做好“零就业家庭”、“4050”人员、登记失业人员、随军家属、大中专毕业未就业人员等群体的就业工作。以实施“阳光工程”为重点，继续加强农村富余劳动力转移就业培训，通过正规培训、特色培训和“订单式”培训等多种模式推进就业，同时切实落实开发商安排本地农民就业的相关约定。

――进一步完善社会保障体系。加大社保扩面征缴力度，强化社保基金管理，确保基金安全。加强企事业单位离退休人员社会化管理服务工作，积极探索解决60岁以上城镇居民养老保险问题。进一步健全被征地农民社会保障机制，切实落实各项社保奖励，带动广大农民参加社会保险。进一步提高新型农村合作医疗人均筹资标准，提高城镇从业人员和城镇居民基本医疗保险待遇，不断完善定点医疗机构考核指标体系和准入制度，提升其服务水平。继续完善医疗便利报销制度，解决好基本医疗保险市外转诊、异地治疗监控难等问题。抓好城乡居民低保工作，建立健全保障标准动态调整机制。

――努力提高城乡居民收入。切实落实职工带薪年休假制度，增加企事业单位职工工资性收入，并根据在职职工收入增加的情况适当提高退休人员基本养老金。切实落实最低工资规定，进一步提高最低工资标准。积极引导乡镇企业发展，支持企业加大技术改造力度，促进企业上规模、上水平，同时高水准地开发乡村旅游项目，打造槟榔村5A级乡村游，培育扶持一批“旅游示范村”、“旅游示范户”，拓宽农民增收渠道。在提高城乡居民收入的基础上，积极培育服务性消费等消费热点，开展家电下乡、农机下乡活动，扩大消费信贷市场，拉动消费需求。

――加强城乡文化建设。进一步加大政府投入力度，建立健全公共文化服务平台，促进文化事业发展。加快推进市群众艺术馆、两区综合文化中心等设施建设，积极开展社区文化活动、乡村文化活动、文化下乡活动，继续实施农家书屋、农村电影放映、广播电视村村通等文化惠民工程，丰富城乡居民的文化生活。继续加强和谐文化建设，广泛开展精神文明创建活动，倡导科学、健康、文明的生活方式。

――保持社会和谐稳定。加大普法工作力度，引导全社会学法、知法、守法、用法，不断提高全民法律素质。充分发挥社区、村居基层管理作用，把调解纠纷、化解矛盾、消除隐患、防范风险的“关口前移”。进一步落实信访责任制，有效预防和妥善处置群体性事件。建立健全外来人口和出租屋管理相关规定，实现流动人口规范化管理。进一步加大社会治安基础投入，不断完善社会治安防控体系，加强社会治安综合治理，严密防控和严厉打击各种犯罪活动。切实落实安全生产责任制，加大安全隐患排查治理工作力度，严厉打击非法建设、非法生产、非法经营行为，坚决遏制重特大安全事故的发生。抓好食品药品质量安全工作，进一步完善认证、检测和监管体系。建立健全重大公共安全事件的预警、应急、救援体系，增强政府应对突发事件的能力。进一步加强国防动员和民兵预备役工作，大力支持国防建设，广泛开展双拥共建活动。继续做好民族、宗教、外事、侨务、统计、新闻、档案、修志、科协、妇女儿童等工作，红十字、老龄和残疾人等事业也要取得新进展。

（六）加强政府自身建设，提升科学发展能力。面对复杂的发展环境和艰巨的工作任务，我们必须全力抓好政府自身建设，适应发展形势的要求。

――加强民主法制建设。坚持依法行政，认真执行市人大及其常委会的决议、决定，自觉接受市人大的法律监督、工作监督和市政协的民主监督，定期向市人大及其常委会报告工作，向市政协通报情况、征询意见。认真听取各民主党派、工商联、无党派人士的意见和建议，充分发挥工会、共青团、妇联等人民团体的桥梁纽带作用。加强电子政务建设，不断完善和切实落实政务公开制度，接受新闻舆论和人民群众对政府机关及其工作人员的监督。进一步深化行政执法体制改革，强化执法人员综合素质建设，严格执行行政执法责任制、评议考核制和过错追究制，规范行政执法行为。

――推进行政管理体制改革。继续做好省里下放行政管理事项的对接和完善工作，进一步深化行政审批制度改革，对审批权限进行全面清理，该取消的取消，该简化的简化，同时加强审批规范化、程序化建设，严格落实工作时限要求，净化审批行为。不断完善海棠湾机构改革并及时总结经验，按照中央和省有关行政管理体制改革的指导精神，立足三亚发展的实际需要，适时推动政府机构改革调整，理顺职、权、责的关系。加快各镇机构改革步伐，强化基层政府的社会管理和公共服务职能。适当提高村居干部待遇，加快探索完善村居干部培训和激励机制，充分调动基层干部的工作积极性。

――切实增强政府执行力。加强执行文化建设，根据三亚经济社会发展目标任务，按照岗位、职权与责任相匹配的原则，给市政府领导以及市政府各部门、各单位科学分配任务，并大力强化工作效能监察和机关绩效考评。不断完善政府行政效能监察办法，增强其程序性、规范性、可行性，有效地将领导、部门和单位的工作效能、工作成绩与考核评价联系起来。坚持定期或不定期召开市政府党组会议，并根据实际情况邀请市人大和市政协有关领导参加，听取市政府领导和部门、单位工作陈述，对工作绩效进行集体分析、公正评判。

――加强政府廉政建设。严格执行廉洁从政各项规定，厉行节约，坚决反对铺张浪费，有效降低行政成本，打造节约型政府。认真贯彻落实公务员法，切实加强对公务员的教育、管理和监督，积极建立健全教育、制度、监督并重的惩治和预防腐败体系，确保权力在阳光下运行。强化政府层级监督，充分发挥监察、审计等专门监督的作用，切实加强土地出让、工程招投标、产权交易、政府采购等重点领域的监管，严肃查处各类违法违纪案件，坚决纠正损害群众利益的不正之风。

各位代表！尽管当前的发展形势仍然比较复杂，但是希望与困难同在，机遇与挑战并存。让我们高举中国特色社会主义伟大旗帜，更加紧密地团结在以胡锦涛同志为总书记的党中央周围，在市委的坚强领导下，万众一心，脚踏实地，开拓创新，锐意进取，为推动三亚又好又快发展，全面实现小康社会而努力奋斗！
